# 3D Morphological Scanning and Environmental Correlates of *Bufo gargarizans* in the Yellow River Basin

**DOI:** 10.3390/ani14030369

**Published:** 2024-01-24

**Authors:** Zihan Li, Xuecheng Guo, Zeguang Guo, Xiaoqin Shi, Jin Zhou, Zhidong Liu, Qi Xiao, Youhua Chen

**Affiliations:** 1 Chengdu Institute of Biology, Chinese Academy of Sciences, Chengdu 610041, China; lizh1@cib.ac.cn (Z.L.); guoxc1@cib.ac.cn (X.G.); guozeguang22@mails.ucas.ac.cn (Z.G.); shixiaoqing21@mails.ucas.ac.cn (X.S.); m18260029036@163.com (J.Z.); liuzd@cib.ac.cn (Z.L.); m18033705433@163.com (Q.X.); 2 University of Chinese Academy of Sciences, Beijing 100049, China

**Keywords:** morphology, three-dimensional measurement, adaptive strategy, *Bufo gargarizans*

## Abstract

**Simple Summary:**

Morphology can give us a better understanding of amphibian adaptations to the environment, which are not well responded to by traditional morphological studies. Therefore, we used 3D scanning technology, combined with the analysis of environmental factors, to reveal the 3D morphological characteristics, spatial variation patterns, and driving factors of *Bufo gargarizans* in the Yellow River Basin. The results showed that the overall body size of *B. gargarizans* tended to become smaller with increasing altitude, confirming that the change in body size did not follow Bergman’s law. We also found larger appendages and more complex bodies in high-elevation populations compared to low-elevation populations. In addition, altitude also had a strong influence on the morphogeometric patterns of *B. gargarizans*, and latitude and temperature was also closely related to their morphogeometric patterns.

**Abstract:**

Morphology plays a crucial role in understanding the intricacies of biological forms. Traditional morphometric methods, focusing on one- or two-dimensional geometric levels, often fall short of accurately capturing the three-dimensional (3D) structure of organisms. The advent of 3D scanning techniques has revolutionized the study of organismal morphology, enabling comprehensive and accurate measurements. This study employs a 3D structured light scanning system to analyze the morphological variations in the Chinese toad (*Bufo gargarizans* Cantor, 1842) along the Yellow River Basin. The 3D digital model obtained from the scan was used to calculate various morphological parameters including body surface area, volume, fractal dimensions, and limb size. The research explores geographic variability patterns and identifies environmental drivers affecting the 3D phenotypic variation of *B. gargarizans*. Results reveal a bimodal pattern of variation in the toad population, with higher elevations exhibiting smaller body sizes, greater appendage proportions, and more complex body structures. Linear regression analyses highlight the influence of elevation and annual mean temperature on the morphological variation of *B. gargarizans*, with elevation playing a significant role. This study underscores the significance of 3D morphometric analysis in unraveling the intricacies of organismal morphology and understanding the adaptive strategies of species in diverse environments.

## 1. Introduction

Morphology is an important branch of biology, that aims to reveal the nature of biological forms [1] and assists for biological classification since the era of Carl Linnaeus. The early morphological measurement mainly includes traditional measurement and geometric measurement. Traditional morphometry focuses on providing a linear description of its size and shape, but the results obtained by this method often do not reflect the original shape well [2]. Geometric morphometrics focuses more on information about the geometric form of the subject, which can make it easier to obtain and analyze data, and makes quantitative morphometric studies more accurate [3]. However, these traditional methods can only characterize morphological variations on a one- or two-dimensional geometrical level. In contrast, the structure of an organism is a three-dimensional (3D) entity existing in space, and these methods inevitably misinterpret the reflection of the original structure, which in turn generates large measurement errors. To better study the structural characteristics of organismal morphology, a more comprehensive and accurate methodology is needed.

In recent years, the development of 3D scanning techniques has provided a new means of studying the morphology of living organisms. The 3D measurement technique is one of the latest techniques used in the field of metrology, by recording the 3D coordinates of many spatial points on the surface of the object being scanned, the spatial data will be obtained to construct a 3D digital model using reverse modeling software. Morphometry based on 3D geometry not only measures the true area and volume of an organism’s surface but also obtains a more realistic picture of the organism’s shape by analyzing morphological features at the 3D level [4,5]. Compared to traditional point and line measurements, 3D data can more comprehensively and accurately capture structural features of organism morphology, identify and quantify the magnitude of morphological differences between populations, and better understand the factors that lead to differences in the morphology of different populations; it can also better reflect the divergence of homologous traits, and with the help of shape analysis, accurately reflect the variations and similarities between populations, which allows for in-depth exploration of taxonomic, evolutionary and phenotypic adaptive features [6,7]. Currently, 3D measurement techniques have been widely used in the fields of mechanical manufacturing, medical imaging, reverse engineering, geographic information systems, and cultural relics protection, however, there are relatively few related studies in the biological field. The current studies mainly focus on the morphological comparison between extant and ancient vertebrates, covering a variety of scientific issues such as skull evolution, comparison of appendicular skeletal morphology, and comparison of important functional organs [8,9,10]. Several studies on early fishes demonstrated the outstanding and accurate ability of 3D measurement techniques in measuring external morphological features and constructing micro details of organisms through 3D reconstruction of fossils [11,12]. Ivanović et al. [13] also used 3D scanning in their studies of the skeletal morphology of *Bufo bufo* and *Bufo spinosus*, demonstrating its usability for the study of amphibian morphology.

Chinese toad (*Bufo gargarizans*, Cantor, 1842) is distributed in most provinces and municipalities in China except Xinjiang, Taiwan, Hong Kong, Macao, and Hainan, and abroad in areas such as the Korean Peninsula and the Russian Far East. As one of the widely distributed amphibians in China, *B. gargarizans* has remarkable morphological variation and rich habitats, and has obvious adaptations to its ecological environment, both in terms of morphological and structural changes, as well as physiological and behavioral adaptations to environmental factors [14,15,16]. It is important to understand the body structure of *B. gargarizans* from a morphological point of view and to analyze its survival strategies and adaptive mechanisms. However, there is still a lack of research on phenotypic adaptations and morphogeometric variation of *B. gargarizans* in the 3D dimension.

Our objectives in this study were: (1) Using a 3D structured light scanning system to acquire 3D spatial point cloud data from freshly prepared specimens of *B. gargarizans* from the Yellow River Basin, and 3D reconstruction was carried out to digitize and preserve the morphological features; (2) Computing the morphological parameters of the 3D digital models of *B. gargarizans*, including the body surface area, body volume, relative body surface area, 3D fractal dimensions, and the relative sizes of appendages, to investigate the geographic variability patterns of the 3D morphology along the Yellow River Basin; (3) Extracting the environmental factors (temperature, climate, precipitation data, etc.) corresponding to each sample, and investigating the drivers of the 3D phenotypic variation of *B. gargarizans* by principal component analysis (PCA), linear regression (LR), redundancy analysis (RDA) and variance partitioning analysis (VPA).

## 2. Materials and Methods

### 2.1. Study Area

The Yellow River originates in Qinghai Province, China, and its basin spans from the west to the east, with complex and varied topography and landscape in different regions. The upstream area consists of high plateaus and mountain ranges, the middle reaches are dominated by hills and basins, and the downstream area is characterized by plains. Vegetation types, climate characteristics, and land use types vary from region to region, spanning arid, semi-arid, and semi-humid climate zones from northwest to southeast; precipitation and distribution are also uneven, with the upstream area receiving less precipitation, mainly in summer, while the middle and downstream areas of the basin are relatively rich in precipitation, concentrating in summer and autumn. Such complex and variable geographical characteristics pose a serious challenge to the survival and adaptation of animals [17,18].

### 2.2. Sampling Method

**Figure 1:**
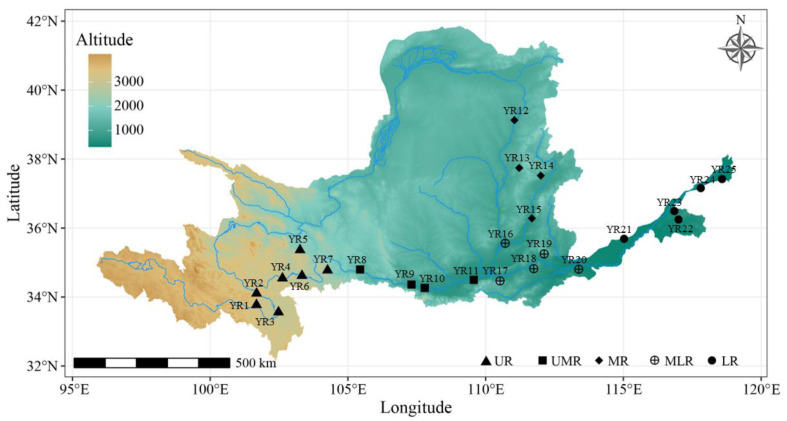
Sampling locations information of *Bufo gargarizans* in the Yellow River Basin.

Concentrated sampling was conducted from April to August 2022, where adult *B. gargarizans* were captured from upstream to downstream along the Yellow River Basin, and in the evening along the pre-set sample line for amphibian surveys, about 1km along the water. In total, 244 adult *B. gargarizans* were collected from 25 sites in the Yellow River Basin (Figure 1). According to the characteristics of the Yellow River Basin and the spatial distance of the sampling sites, each sampling site was grouped into populations (Upper Reaches, UR; Upper-Middle Reaches, UMR; Middle Reaches, MR; Middle-Lower Reaches, MLR and Lower Reaches, LR). Individual toads collected in the field were immediately numbered and brought back indoors to be measured by vernier calipers in a dissecting tray. Individual toads were killed by double demyelination, fixed in formalin solution for about 1 h until the morphology was stable, and then scanned in three dimensions, and the scanned point cloud data were encapsulated in high detail to generate a 3D model that was saved as a engineering file for the subsequent measurements of 3D indices. During the sample collection process, each collected sample was recorded with a GPS instrument to locate the latitude, longitude, and elevation of the corresponding sampling site data.

### 2.3. Acquisition of 3D Morphological Data

#### 2.3.1. 3D Scanning Platform Building

In this study, a portable 3D structured light scanning system (Hangzhou Shining 3D Company, Hangzhou, China, including the 3D scanner EinScan Pro 2X Plus 2020 and the 3D data processing software EXScan Pro v3.6.0.5) was used, and the main body and the working module are shown in Figure S1. Under the same reference frame, the 3D binocular structured light scanner was used to scan the toad specimen in fixed mode and obtain the 3D morphological data of the specimen. The system was calibrated before use to correct for errors, and during the scanning process, it was ensured that the position of the specimen was the same after each change of specimen, while keeping the scanner parameters constant and the surrounding light conditions roughly unchanged. After the scanning process is completed, multiple point cloud data are obtained, and these segmented point cloud data have common data parts, with the help of which the complete point cloud data can be generated. The point cloud data were reconstructed using EXScan Pro v3.6.0.5 software to obtain a complete 3D model. The 3D structured light scanner was used to obtain the 3D spatial point cloud data of *B. gargarizans*, and then the excess noise removal and high detail encapsulation were carried out to generate a highly accurate 3D model of *B. gargarizans*, which can be used for the subsequent measurement of various morphological parameters.

#### 2.3.2. 3D Morphological Feature Quantization

With the deepening of the study of biological morphology, it is no longer possible to use simple regular geometry to replace animal shapes with different shapes in reality. The morphological parameters of research objects can be obtained through 3D digital models, and these accurate parameters can meet the basic requirements of numerical simulation software for its motion process or environment adaptation. In this study, several 3D morphological parameters were applied to the morphological measurement of *B. gargarizans*. After 3D scanning and processing software, 3D data such as surface area and volume of limbs and body of *B. gargarizans* specimens were extracted to explore how these parameters reflected morphological variation of different geographical populations of *B. gargarizans.*

In the 3D scanning system, the 3D digital model of *B. gargarizans* is determined based on the triangular mesh point matrix by the triangulation method. The inverse modeling software converts the point cloud data into a mesh consisting of many small triangles on the surface, a process known as triangulation of the point cloud data. The body surface area (BSA) of *B. gargarizans* is the sum of the areas of all the triangles, and the body volume (BV) is equal to the size of the space occupied by the closed figure enclosed by all the triangles. Accordingly,

(1) $$BSA=\frac{\iint }{\sum }ds=\underset{n\to \infty }{\lim}\sum_{i=1}^{n}{∆S}_{i}\;$$

(2) $$BV=\frac{∭}{\Omega }dv=\underset{\lambda \to 0}{\lim}\sum_{i=1}^{n}{∆v}_{i}\;$$

where the integral in Equation (1) is based on a limit containing the sum of an infinite number of cells, each of which represents a small portion of the two-dimensional plane *S_i_* in the three-dimensional Euclidean coordinate system. As the region element *S_i_* becomes sufficiently small, the summation of *S_i_* yields the surface area to a very high degree of accuracy. Equation (2) obtains the true volume of the object by summing the contributions from each small closed region *v_i_* and as the maximum diameter *λ* of each closed region becomes small enough to approach infinity, at which point the true volume of the object is obtained.

Relative body surface area (RBA) expresses the total area per unit volume of an object, which is defined as,

(3) $$RBA=\frac{BSA}{BV}$$

Fractal dimension (*D_f_*) is an important parameter used to quantitatively describe very complex fractal objects, which are usually non-smooth, irregular, broken, and other extremely complex fractal objects. *D_f_* characterizes the complexity and roughness of the fractal body, i.e., the larger the *D_f_* is, the more complex and rougher the object is, and the more difficult it is to approximate it with smooth and regular geometry, and vice versa. Since this study applies the point cloud data obtained from 3D scanning, it focuses on the 3D *D_f_*. Using the derivation of the island method proposed by Mandelbrot et al. [19], *D_f_* is estimated as,

(4) $$D_{f}=3\frac{\ln BSA}{\ln BV}$$

Other 3D morphometric parameters used in this study included forelimb surface area (FSA), forelimb volume (FV), relative forelimb size (FV/BV), hindlimb surface area (HSA), hindlimb volume (HV) and relative hindlimb size (HV/BV). In addition, the following 1D morphological data were measured in the *B. gargarizans* samples. The specific variables and their biological significance are shown in Table 1.

### 2.4. Data Analysis

Firstly, we compared the morphological variation of *B. gargarizans* in different geographic populations along the Yellow River Basin using 1D and 3D morphological data. After that, we used the ‘*prcomp*’ function of R [20] to conduct PCA to investigate the variation of different geographic populations of *B. gargarizans* along the Yellow River Basin, and verified the superiority of the 3D morphometric data compared with the 1D morphometric data in reflecting the morphological differences. Morphological differences between populations were verified using one-way analysis of variance (ANOVA) and Tukey’s HSD tests, and differences in morphology between males and females within the same population were verified using *t*-tests.

To investigate the influence mechanisms of bioclimatic variables and environmental factors, such as spatial factors and net primary productivity (NPP), on the morphogeometric variability of *B. gargarizans* in the Yellow River Basin, 19 bioclimatic variables and elevation datasets were downloaded from the WorldClim database (https://worldclim.org/, accessed on 1 June 2022), and NPP datasets were downloaded from the website of the Center for Resource and Environmental Science and Data of the Chinese Academy of Sciences (https://www.resdc.cn/, accessed on 1 June 2022). The downloaded bioenvironmental data were imported into Arcgis 10.5 (ESRI, Redlands, CA, USA), and the corresponding environmental factors were extracted based on the latitude and longitude coordinates of each sample. To reduce multicollinearity among environmental variables, the Spearman correlation coefficient between each pair of environmental variables was calculated. When collinearity was eliminated, one representative factor of correlation |r| ≥ 0.7 was retained. After the screening, the final set of 10 environmental factors used for follow-up analyses was: temperature factors including Annual Mean Temperature (BIO1), Mean Diurnal Range (BIO2), and Min Temperature of Coldest Month (BIO6), the precipitation factors including Annual Precipitation (BIO12), Precipitation of Driest Month (BIO14), and Precipitation Seasonality (BIO15), and the spatial factors including longitude (LON), latitude (LAT), and elevation (ELE), and NPP.

The ‘*rda*’ function and ‘*varpart*’ function in the ‘vegan’ package were used to complete the RDA and VPA, respectively, to validate the relationship between 1D morphology and 3D morphological variation of *B. gargarizans* in the Yellow River Basin [21]. All data were normalized using the ‘*scale*’ function for analysis.

## 3. Results

### 3.1. PCA of Morphological Data

**Figure 2:**
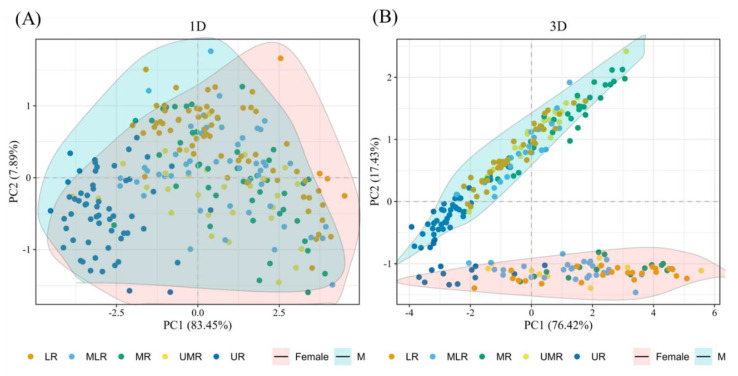
Results of principal component analysis of *Bufo gargarizans* sexual differences reflected by (**A**) 1D and (**B**) 3D morphology data. 1D, one-dimensional; 3D, three-dimensional.

PCA of 1D and 3D morphological data of *B. gargarizans* of different sexes showed that the data of both dimensions had a high interpretation rate (Figure 2). The scores of two principal components obtained from the analysis of the 3D morphological data accounted for 93.85% of the variation, which was higher than that of the 1D morphological data (91.34%). It was able to better differentiate between male and female individuals.

**Figure 3:**
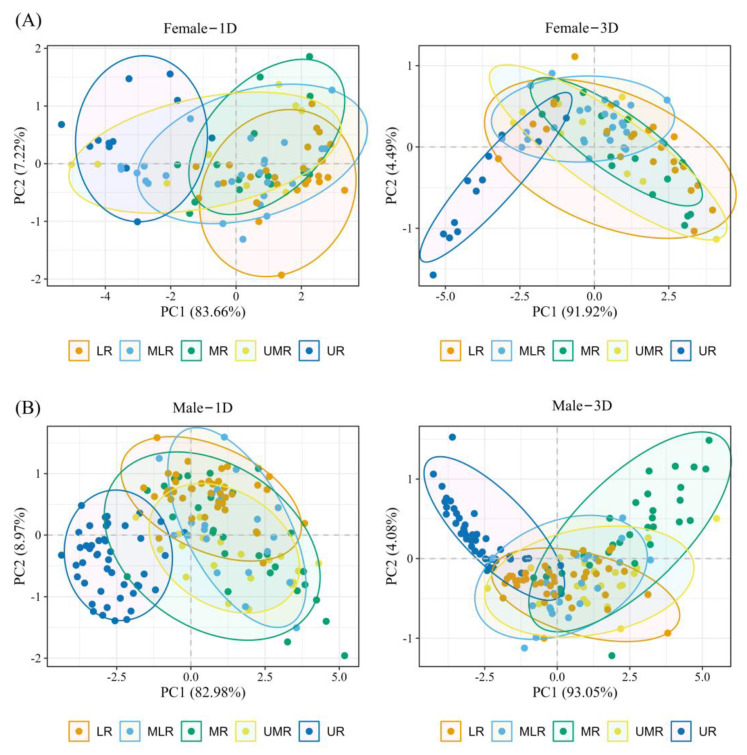
Results of PCA of *B. gargarizans* in lower reaches (LR), middle and lower reaches (MLR), middle reaches (MR), upper and middle reaches (UMR), and upper reaches (UR) populations in the Yellow River Basin. (**A**) Female populations; (**B**) Male populations.

Further PCA analyses by geographic population segmentation revealed that the explanatory rates of the two principal components obtained from the 3D morphological data were higher than those obtained from the 1D morphological data for both sexes and that the morphology of the UR population showed a clear distinction from the other populations (Figure 3).

### 3.2. Patterns of Changes in 1D and 3D Morphology

**Figure 4:**
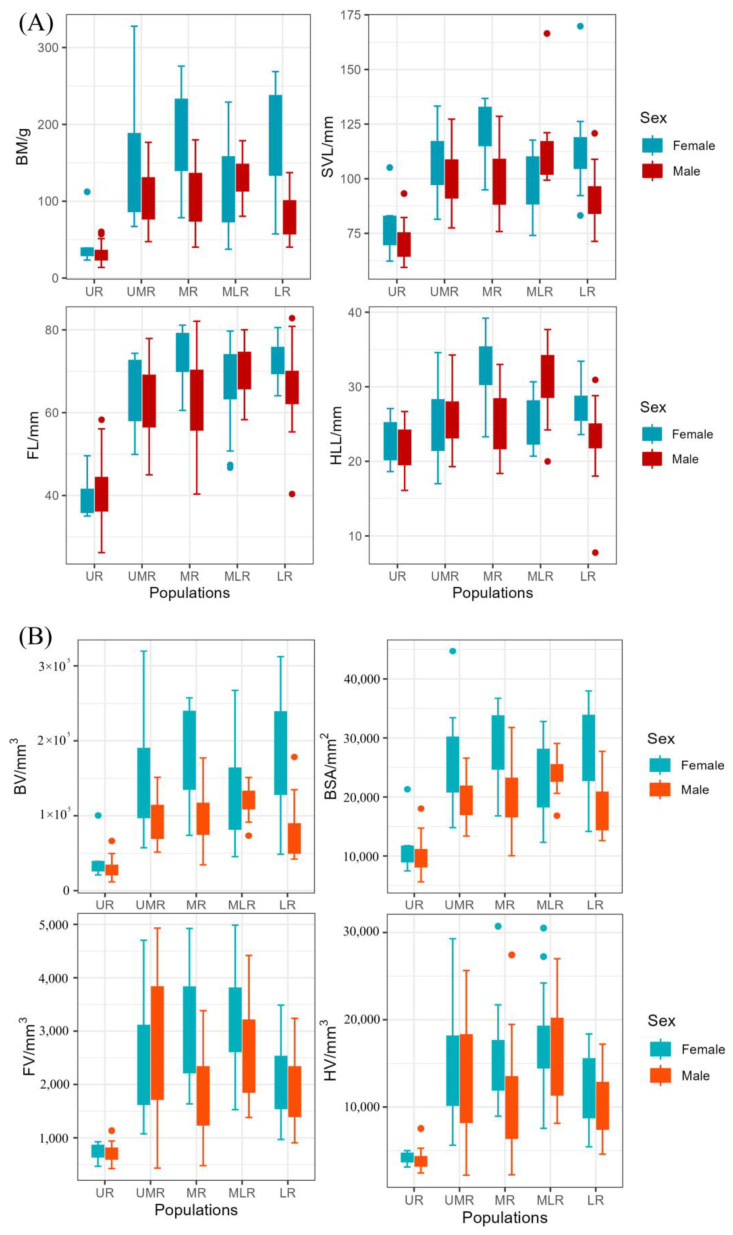
Patterns of (**A**) 1D and (**B**) 3D morphological changes in *B. gargarizans* in the Yellow River Basin, of which the meanings of the abbreviations are given in Section 2.3 and Table 1.

Combining the morphological data of different geographic populations, we found that the 1D and 3D morphology of *Bufo gargarizans* showed a trend of increasing and then decreasing overall body size along the Yellow River Basin. The morphology of toads in the UR population at high altitudes was the smallest, the range of variation in body weight was the largest in the UMR population, and the range of variation in head and body length was the largest in the MR population. The 1D morphology of the MLR population was between the MR and LR populations. *B. gargarizans* population in the LR had a large difference between the sexes in body size, with females significantly larger than males (Figure 4).

**Figure 5:**
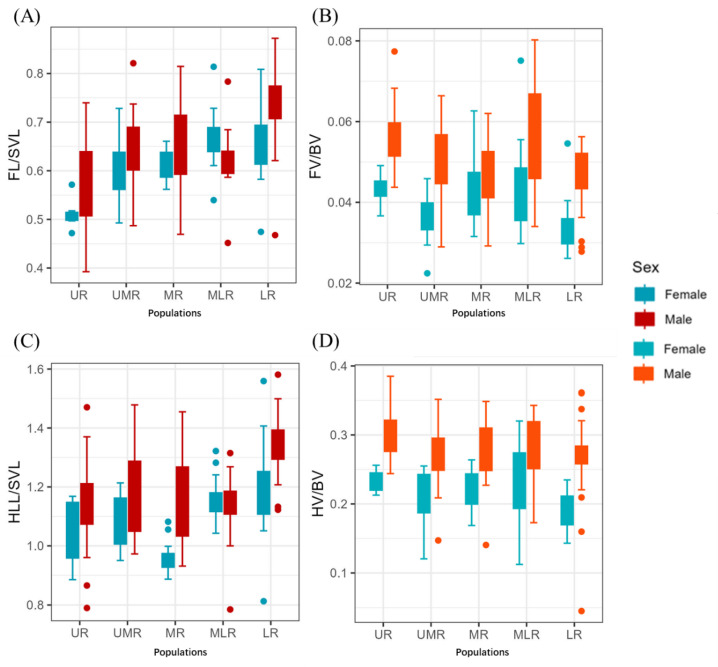
Patterns of appendage variation in populations of *B. gargarizans* in the Yellow River Basin. (**A**) relative forelimb length, (**B**) relative forelimb volume, (**C**) relative hindlimb length, and (**D**) relative hindlimb volume.

In this study, the relative forelimb length of *B. gargarizans* gradually became larger as the watershed moved from upstream to downstream, but the relative forelimb size gradually became smaller as the watershed moved from upstream to downstream, and the female population was smaller than that of the male population; the relative hindlimb length and the relative hindlimb size varied roughly in the same way as that of the forelimb as the population moved along the watershed (Figure 5).

### 3.3. Patterns of Change in Relative Body Surface Area and 3D Fractal Dimension

**Figure 6:**
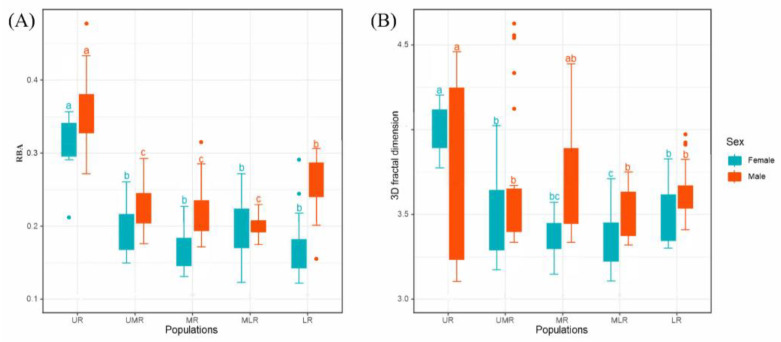
Comparison of (**A**) the relative body surface area and (**B**) the 3D fractal dimension of various groups of *B. gargarizans* in the Yellow River basin. RBA is relative body surface area. Different lower case letters indicate significant differences between populations (*p* < 0.05).

Fitted curves with body volume and surface area statistics of *B. gargarizans* in the Yellow River Basin showed that males had smaller relative surface areas compared to females, suggesting that *B. gargarizans* have different growth rates of surface area and body size in different sexes (Figure 6A). The slopes of the volume-surface-area fitted curves for females and males of the five geographic populations were further plotted, indicating a general downward trend (Figure S2). Populations at lower elevations had the smallest RBA and a reduced proportion of limbs, and mid- and down-stream populations showed morphological characteristics that approximated those of downstream populations. However, mid and lower reaches toads, especially males, were smaller relative to females, and there was a large size difference between males and females.

**Figure 7:**
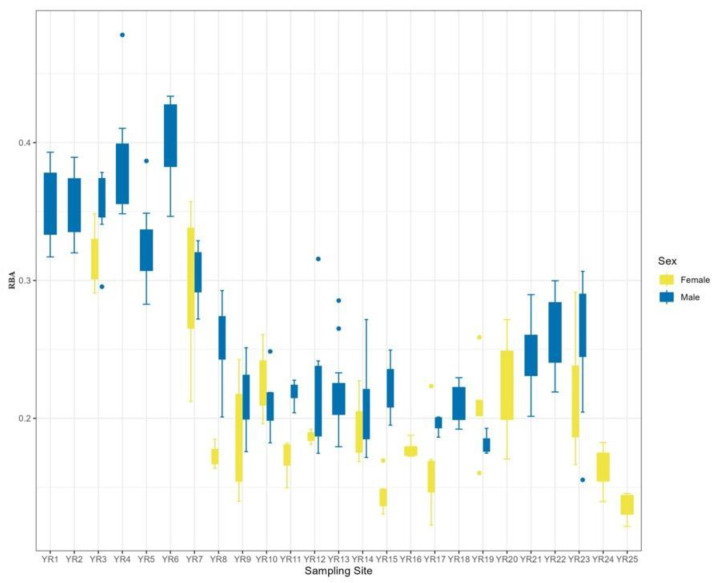
Patterns in relative body surface area variation in male and female *B. gargarizans* in the Yellow River Basin. RBA is relative body surface area.

At the sampling point level, the RBA decreased gradually as the sampling point approached downstream, but the RBA in the midstream area was close to the value in the downstream area, indicating that the toads in both areas were larger in size. At the sex level, females were all larger than males at the same sampling points (Figure 7).

Calculated according to Equation (4), the 3D fractal dimension statistics of various populations are shown in Table 2, and the morphological complexity characterized by 3D fractal dimensions is higher in different geographic populations (Figure 6B). In addition, it can be seen that the male population in the middle reaches has the highest 3D fractal dimension, and the female population in the middle reaches has the lowest value of 3D fractal dimension (Figure S3). The results of the 3D fractal dimension indicate that the sex of *B. gargarizans* in the middle reaches has the highest variability of the 3D fractal dimension.

**Figure 8:**
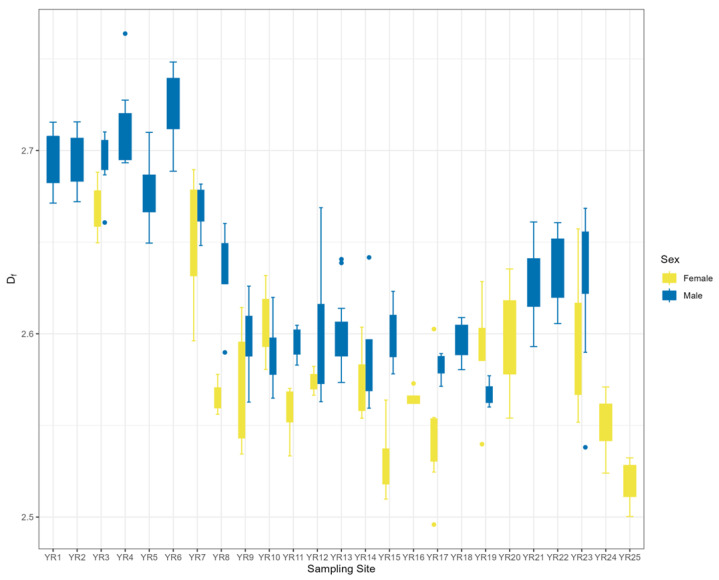
Patterns of 3D fractal dimension variation in male and female *B. gargarizans* at the sample site level in Yellow River Basin.

At the sampling point level, the decreasing fractal dimension from upstream to downstream indicated that *B. gargarizans* body size complexity was decreasing. In terms of sex differences, the degree of variability in 3D fractal dimensions was highest in the midstream, where the difference between males and females was most pronounced (Figure 8).

### 3.4. Drivers of 3D Morphological Variability

**Figure 9:**
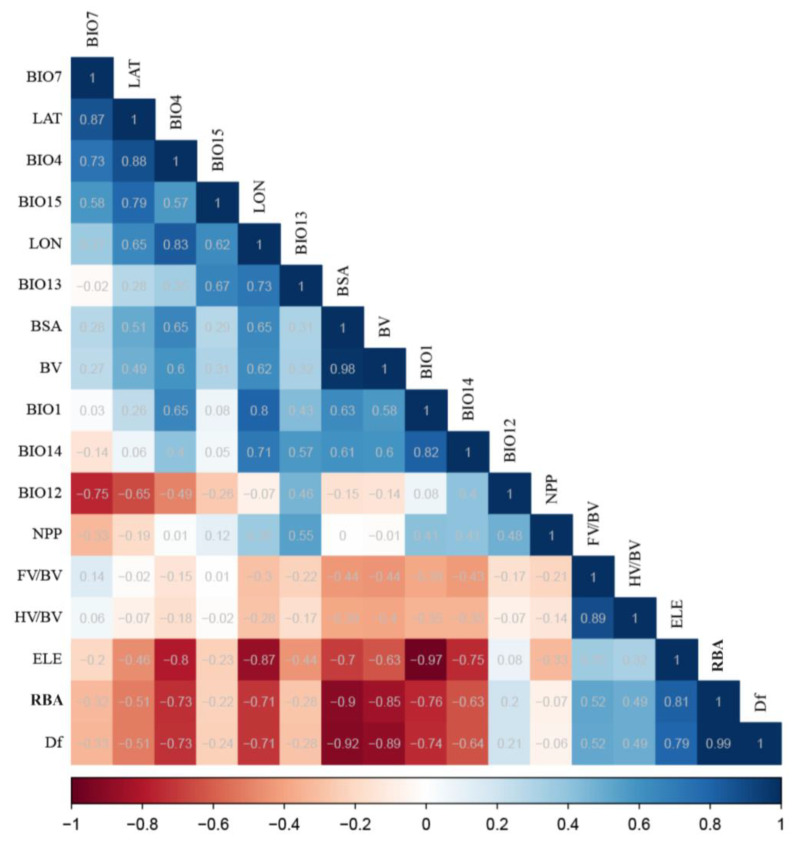
Correlation analysis of morphological variables and environmental variables.

Correlation and regression analyses of morphological variables with environmental as well as spatial variables of *B. gargarizans* showed that elevation (ELE) had strong positive correlations with BSA and BV, and strong negative correlations with RBA and 3D fractal dimension (Figure 9). Annual mean temperature (BIO1) was also significantly correlated with BM, SVL, the relative size of appendages (FV/BV, HV/BV), and RBA, whereas, net primary productivity (NPP) and annual precipitation (BIO12) both had low correlations with morphological variables.

**Figure 10:**
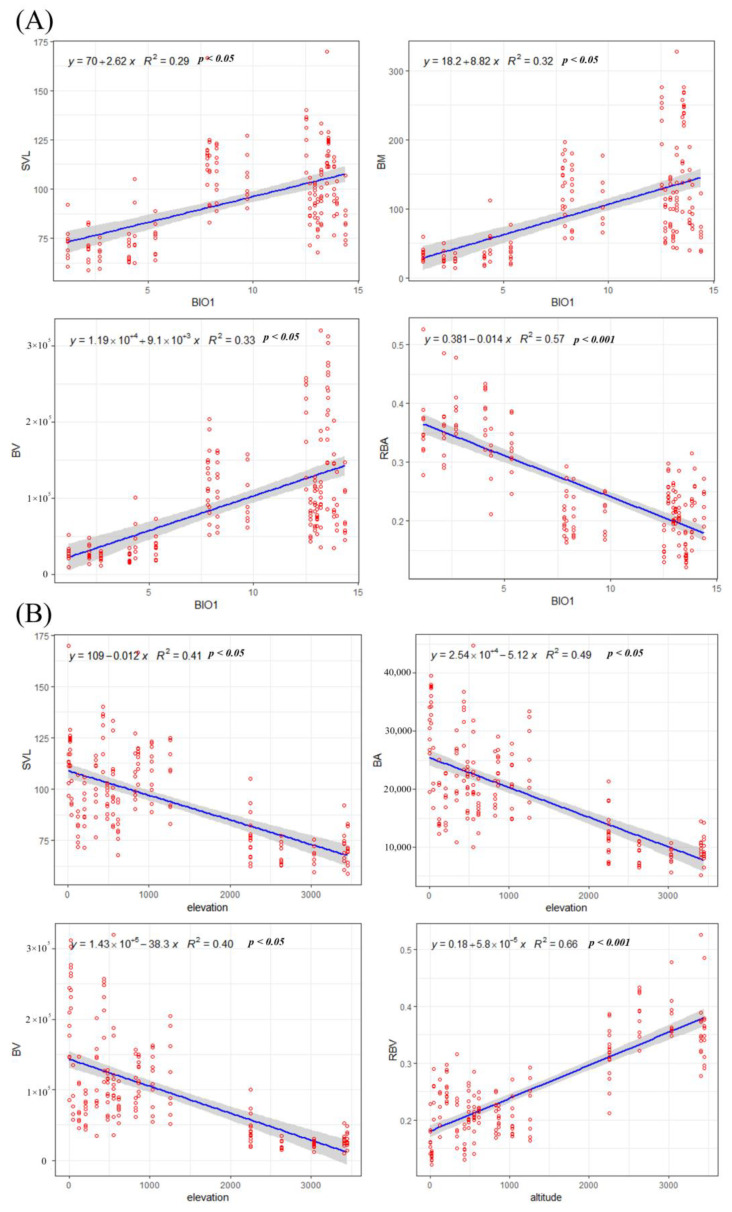
Linear regression modeling of (**A**) BIO1 and (**B**) ELE with SVL, BM, BV, and RBA. The meanings of the abbreviations are given in Section 2.3 and Table 1. Gray areas represent 95% confidence intervals.

After establishing one-way linear regression equations and models for BIO1, ELE, and NPP, and using gray-shaded bands on both sides of the regression line to indicate the size of confidence intervals (*p* < 0.05), it was found that, for BIO1, the results showed that the trend of morphological size change of *B. gargarizans* was to increase with the increase of the annual mean temperature (Figure 10A). For ELE, the change in morphology with elevation showed a trend consistent with the effect of temperature (Figure 10B), in which the change in RBA in response to the increase in elevation was highly significant. For NPP, no correlation was shown with morphology (Figure S4).

**Figure 11:**
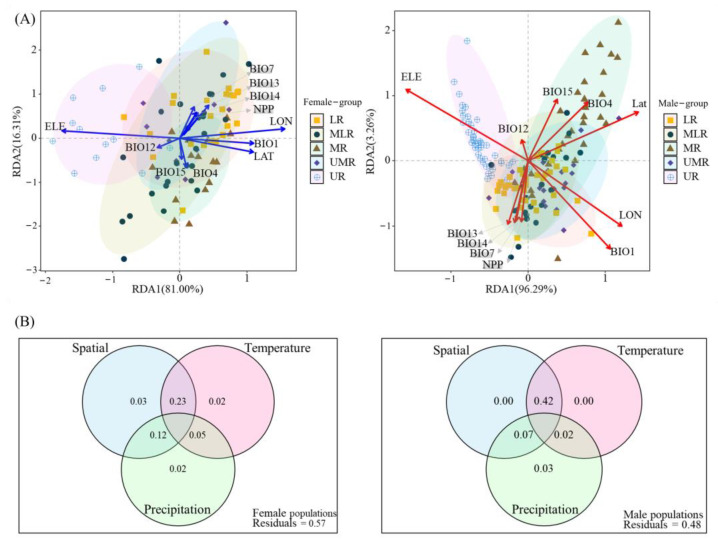
(**A**) Redundancy analysis of environmental and geographic factors on the sexual dimorphism of *B. gargarizans*; (**B**) Venn diagram based on the variance decomposition analysis of 3D shape variation of *B. gargarizans* by space, temperature, and precipitation. Red and blue vectors represent environmental variables.

The results of RDA and VPA indicated that the 3D morphological variation of *B. gargarizans* was most affected by altitude, with a highly negative correlation, and that high-altitude populations were significantly differentiated from the other populations; longitude had the second largest effect on the 3D morphological variation, and the 3D morphology increased with increasing longitude; the contributions of mean annual temperature and latitude were of comparable magnitude in *B. gargarizans* populations; and the effects of precipitation and seasonal temperature changes on the variation of morphology were relatively small (Figure 11).

## 4. Discussion

Our study showed that the overall trend of morphogeometric variation of *B. gargarizans* in the Yellow River Basin showed that from upstream to downstream, the body size became larger (Figure 4), the relative size of the appendages (Figure 5), the relative body surface area and the 3D fractal dimension gradually decreased (Figure 6). Most of the 3D data reflected a bimodal pattern of variation from upstream to downstream in the population of *B. gargarizans* in the Yellow River Basin. *B. gargarizans* at higher elevations had relatively smaller body volume, shorter snout-vent length, and larger relative body surface area. In the pattern of appendage variation, *B. gargarizans* at higher elevations had shorter but larger appendages and those at lower elevations had longer but smaller appendages. The decrease in the 3D fractal dimension also suggests that the decrease in elevation from the upper to the lower reaches of the Yellow River Basin resulted in a decrease in the complexity of the body shape of *B. gargarizans* and a smoother body surface.

Bergmann’s rule [22] recognizes that animals living at lower temperatures have larger body sizes, but this conclusion generally applies to endotherms, and its applicability to ectotherms has been controversial. Previous studies have reached very different conclusions: A pattern of increasing body size with increasing elevation exists in many terrestrial breeding frogs (*Strabomantidae*) living in the Andean region [23], and a similar pattern of morphological change has been found in *Bufo minshanicus* from the eastern Tibetan Plateau [24]. However, the studies by Adams et al. [25] and Jiang et al. [26] did not find a correlation between body size and temperature in amphibians. The results of our study are the same as the latter’s conclusions, that is, the pattern of body size variation in *B. gargarizans* does not follow Bergmann’s rule. The UR population investigated in this study is located at a high elevation (mean elevation was 2946 m), and it is hypothesized that the increase in elevation has led to a reduction in suitable ecological niches where larger individuals may have difficulty surviving due to insufficient food supply, and thus this cold, arid environment has led to individuals allocating energy used for body growth to other processes [27,28]. This, combined with the fact that *B. gargarizans* migrate to terrestrial habitats after a brief reproductive period in the water [29] and that the overall cold temperatures at high altitudes result in a shorter average activity time, has collectively led to the reduction in the size of the toad and the homogenization of the size of both males and females. This result makes sense because, from another perspective, a larger body size in warmer environments can give it a greater advantage in the competition for survival, predation, and reproduction [30,31].

This was also verified in our linear regression of environmental variables against morphological variation. The body size of *B. gargarizans* increased with increasing annual mean temperature and decreased with increasing elevation (Figure 10). However, comparing the R^2^ of the linear regression models of the two environmental variables with the various morphological indicators, elevation had a stronger explanation for the morphological variation of *B. gargarizans*. This suggests that elevation, as a complex environmental variable, contains environmental characteristics such as low oxygen, high ultraviolet-B (UV-B) radiation, and low precipitation [32,33], which may also contribute to the variation in the morphology of *B. gargarizans* along the elevation distribution to a certain extent. Compared with the existing studies on the effects of UV-B radiation and precipitation on amphibian body size, however, our study showed inconsistent trends [31,34]. This may be related to the habitat characteristics of the Yellow River Basin and the species’ own characteristics and life histories.

The life strategy of morphological variation of *B. gargarizans* under different geographic and environmental conditions can vary. In this study, we mainly explored the variation along the Yellow River Basin in three aspects: size of appendages, relative body surface area, and 3D fractal dimension. With decreasing elevation, the appendages of *B. gargarizans* decreased as a proportion of their relative body size despite increasing in length. This suggests that populations at lower elevations resisted the burden of locomotion and body support imposed by increasing body weight by increasing the length of appendages, whereas populations at higher elevations resisted the complex terrain at higher elevations by increasing appendage size as a proportion of body size [35,36]. In terms of sex differences, males had larger appendages than females in both length and relative size. This finding is consistent with previous studies, where Liao et al. [37] found that males of *Bufo andrewsi* have higher muscle mass, contributing to more stable immobilization of females in amplexus and a greater advantage when confronting conspecific males. In terms of relative body surface area, populations at lower altitudes have a smaller relative body surface area and fewer limbs as a proportion of the body, which is consistent with the previous results on body size variation. In general, larger body size is associated with greater oxygen demand and higher requirements for heat and water retention [38,39,40]. A smaller relative body surface area reduces heat loss and thus better body temperature maintenance, and also reduces water loss. This is a trade-off between growth and survival.

The 3D fractal dimension is a mathematical tool for describing the complexity of 3D fractal geometric objects, allowing for the quantification of the differences between different objects at a relatively subtle level. This indicator has a wide range of applications in areas such as the natural sciences and economic and social activities, such as materials science, geology, and biology [41]. It can be used as a single measure to describe the complexity of suitable habitat for populations that require long-term ecological monitoring, and in combination with the associated spatial scales can be used to explain the effects of these variables on the structure of population assemblages [42,43]. 3D fractal dimensions are also frequently used in the biomedical field to quantify the microstructure of tissues and organs and the extent of lesions [44,45]. In this study, we introduced 3D fractal dimensions to assess the geometric complexity of the body surface of *B. gargarizans*. Since the body surface of *B. gargarizans* is very rough and difficult to be approximated by smooth geometry, the complexity of its spatial 3D morphology can be better characterized by using the 3D fractal dimension. As the elevation decreases, the 3D fractal dimension of *B. gargarizans* gradually decreases, indicating that the adaptation to different environments has led to morphological convergence among different geographic populations. The smoother body surface may imply that the more favorable environment at lower elevations reduces competitive and survival pressures on *B. gargarizans*, or, consistent with previous findings, that the larger body size and smaller relative body surface area cause the skin of *B. gargarizans* to stretch. The reasons for this need to be further investigated and refined.

## 5. Conclusions

In conclusion, we used the 3D morphological data of 244 *B. gargarizans* collected within 25 sample sites in the Yellow River Basin to reveal the 3D morphological characteristics and spatial patterns of change as well as the driving factors of *B. gargarizans* in the Yellow River Basin. Our results found that the overall body size of *B. gargarizans* showed a trend of becoming smaller with increasing elevation, confirming that the body size changes of *B. gargarizans* do not follow Bergmann’s rule. We also found that high-elevation populations have larger appendages and greater body complexity relative to low-elevation populations. In addition, elevation has a stronger influence on the morphogeometric pattern of *B. gargarizans*, and latitude and temperature are also closely related.

## Figures and Tables

**Table 1:** Basic parameters of one-dimensional morphology.

Parameters	Definition/Abbreviation	Unit
Body Mass	BM	g
Snout-Vent Length	SVL	mm
Head Width	HW	mm
Head Length	HL	mm
Forelimb Length	FL	mm
Relative Forelimb Length	FL/SVL	/
Hindlimb Length	HLL	mm
Relative Hindlimb Length	HLL/SVL	/

**Table 2:** Statistics of 3D fractal dimension of different geographical populations.

No.	D_f_	R^2^
UR-Female	2.67	0.99
UMR-Female	2.61	0.99
MR-Female	2.55	0.96
MLR-Female	2.57	0.89
LR-Female	2.54	0.99
UR-Male	2.70	0.98
UMR-Male	2.61	0.96
MR-Male	2.73	0.96
MLR-Male	2.60	0.97
LR-Male	2.63	0.96

## Data Availability

The data are not publicly available due to privacy or ethical restrictions.

## Supplementary Materials

The following supporting information can be downloaded at: https://www.mdpi.com/article/10.3390/ani14030369/s1, Figure S1: Three-dimensional scanner EinScan Pro 2X Plus 2020 work modules. (a) Handheld Module (b) Texture Module (c) Engineering Module, Stepper Motorized Rotary Table Triangle Stand; Figure S2: Relationship between body surface area and volume in females (left) and males (right) of different geographic populations of *B. gargarizans* in the Yellow River Basin; Figure S3: 3D fractal dimension map of females (left) and males (right) in different geographical populations of *B. gargarizans* in the Yellow River Basin; Figure S4: Linear regression modeling of NPP with SVL, BM, BV and RBA.

## References

[1] Lauder G.V. (1981). Form and function: Structural analysis in evolutionary morphology. Paleobiology.

[2] Milroy M.J., Weir D.J., Bradley C., Vickers G.W. (1996). Reverse engineering employing a 3D laser scanner: A case study. Int. J. Adv. Manuf. Technol..

[3] Bernal V. (2007). Size and shape analysis of human molars: Comparing traditional and geometric morphometric techniques. HOMO.

[4] Cox P.G., Hautier L. (2015). Evolution of the Rodents: Advances in Phylogeny, Functional Morphology and Development.

[5] Marlow P.J., Todorović D., Anderson B.L. (2015). Coupled computations of three-dimensional shape and material. Curr. Biol..

[6] Adams D.C., Rohlf F.J., Slice D.E. (2004). Geometric morphometrics: Ten years of progress following the ‘revolution’. Ital. J. Zool..

[7] Miller J.S., Burdick J.A. (2016). Special Issue on 3D Printing of Biomaterials. ACS Biomater. Sci. Eng..

[8] Wang H., Zeng L., Yin C. (2002). A video tracking system for measuring the position and body deformation of a swimming fish. Rev. Sci. Instruments.

[9] Bai M., Yang X. (2014). A review of three-dimensional (3D) geometric morphometrics and its application in entomology. Acta Entomol. Sin..

[10] Davies T.G., Rahman I.A., Lautenschlager S., Cunningham J.A., Asher R.J., Barrett P.M., Bates K.T., Bengtson S., Benson R.B.J., Boyer D.M. (2017). Open data and digital morphology. Proc. R. Soc. B Biol. Sci..

[11] Jerve A., Qu Q., Sanchez S., Blom H., Ahlberg P.E. (2016). Three-dimensional paleohistology of the scale and median fin spine of *Lophosteus superbus*(Pander 1856). PeerJ.

[12] Hou Y., Cui X., Canul-Ku M., Jin S., Hasimoto-Beltran R., Guo Q., Zhu M. (2020). ADMorph: A 3D Digital Microfossil Morphology Dataset for Deep Learning. IEEE Access.

[13] Ivanović A., Cvijanović M., Vučić T., Arntzen J.W. (2022). Differentiation of skull morphology and cranial kinesis in common toads. Org. Divers. Evol..

[14] Liao W.B., Luo Y., Lou S.L., Lu D., Jehle R. (2016). Geographic variation in life-history traits: Growth season affects age structure, egg size and clutch size in Andrew’s toad (*Bufo andrewsi*). Front. Zool..

[15] Yu T., Guo Y., Lu X. (2010). Habitat selection of Asiatic toad, Bufo gargarizans (Cantor, 1842), in southwestern China. Russ. J. Ecol..

[16] Bókony V., Üveges B., Verebélyi V., Ujhegyi N., Móricz M. (2019). Toads phenotypically adjust their chemical defences to anthropogenic habitat change. Sci. Rep..

[17] Wang Y., Tan D., Han L., Li D., Wang X., Lu G., Lin J. (2021). Review of climate change in the Yellow River Basin. J. Desert Res..

[18] Fu G., Chen S., Liu C., Shepard D. (2004). Hydro-Climatic Trends of the Yellow River Basin for the Last 50 Years. Clim. Chang..

[19] Mandelbrot B.B., Passoja D.E., Paullay A.J. (1984). Fractal character of fracture surfaces of metals. Nature.

[20] R Core Team (2023). R: A Language and Environment for Statistical Computing.

[21] Oksanen J., Blanchet F.G., Friendly M., Kindt R., Legendre P., Mcglinn D., Minchin P.R., O’hara R., Simpson G.L., Solymos P. (2019). ‘Vegan’: Community Ecology Package. https://www.mcglinnlab.org/publication/2019-01-01_oksanen_vegan_2019/.

[22] Bergmann C. (1848). Über die Verhältnisse der Wärmeökonomie der Thiere zu ihrer Grösse.

[23] von May R., Lehr E., Rabosky D.L. (2018). Evolutionary radiation of earless frogs in the Andes: Molecular phylogenetics and habitat shifts in high-elevation terrestrial breeding frogs. PeerJ.

[24] Yu T.L., Wang D.L., Busam M., Deng Y.H. (2019). Altitudinal variation in body size in Bufo minshanicus supports Bergmann’s rule. Evol. Ecol..

[25] Adams D.C., Church J.O. (2007). Amphibians do not follow Bergmann’s rule. Evolution.

[26] Jiang Y., Zhao L., Luan X., Liao W. (2022). Geographical Variation in Body Size and the Bergmann’s Rule in Andrew’s Toad (*Bufo andrewsi*). Biology.

[27] Bensakhri Z., Bensouilah S., Zebsa R., Youcefi A., Amari H., Zouaimia A., Lazli A., Houhamdi M., Khelifa R. (2022). Trends to adaptation of the Sahara frog (*Pelophylax saharicus*) larvae across an environmental gradient. Biologia.

[28] Lehr E., Catenazzi A. (2009). A new species of minute Noblella (Anura: Strabomantidae) from southern Peru: The smallest frog of the Andes. Copeia.

[29] Sung H.-C., Park O.-H., Kim S.-K., Park D.-S., Park S.-R. (2007). Abundance and breeding migration of the Asian Toad (*Bufo gargarizans*). J. Ecol. Environ..

[30] Valenzuela-Sánchez A., Cunningham A.A., Soto-Azat C. (2015). Geographic body size variation in ectotherms: Effects of seasonality on an anuran from the southern temperate forest. Front. Zool..

[31] Fu L., Wang X., Yang S., Li C., Hu J. (2022). Morphological Variation and Its Environmental Correlates in the Taihangshan Swelled-Vented Frog across the Qinling Mountains. Animals.

[32] Tang X., Xi L., Niu Z., Jia L., Bai Y., Wang H., Ma M., Chen Q. (2022). Does a Moderately Warming Climate Compensate for the Negative Effects of UV-B Radiation on Amphibians at High Altitudes? A Test of *Rana kukunoris* Living on the Qinghai–Tibetan Plateau. Biology.

[33] Bancroft B.A., Baker N.J., Searle C.L., Garcia T.S., Blaustein A.R. (2008). Larval amphibians seek warm temperatures and do not avoid harmful UVB radiation. Behav. Ecol..

[34] Reniers J., Brendonck L., Roberts J.D., Verlinden W., Vanschoenwinkel B. (2015). Environmental harshness shapes life-history variation in an Australian temporary pool breeding frog: A skeletochronological approach. Oecologia.

[35] Citadini J.M., Brandt R., Williams C.R., Gomes F.R. (2018). Evolution of morphology and locomotor performance in anurans: Relationships with microhabitat diversification. J. Evol. Biol..

[36] Gomes F.R., Rezende E.L., Grizante M.B., Navas C.A. (2009). The evolution of jumping performance in anurans: Morphological correlates and ecological implications. J. Evol. Biol..

[37] Liao W.B., Wu Q.G., Barrett K. (2012). Evolution of sexual dimorphism in the forelimb muscles of Andrew’s toad (Bufo andrewsi) in response to putative sexual selection. Anim. Biol..

[38] Newman R.A., Dunham A.E. (1994). Size at Metamorphosis and Water Loss in a Desert Anuran (*Scaphiopus couchii*). Copeia.

[39] Gillooly J.F., Gomez J.P., Mavrodiev E.V., Rong Y., McLamore E.S. (2016). Body mass scaling of passive oxygen diffusion in endotherms and ectotherms. Proc. Natl. Acad. Sci. USA.

[40] Senzano L.M., Andrade D.V. (2018). Temperature and dehydration effects on metabolism, water uptake, and the partitioning between respiratory and cutaneous evaporative water loss in a terrestrial toad. J. Exp. Biol..

[41] Wang S., Li Z., Zou G., Ma D. (2007). Fractal Characteristics of Gradation Particles in Asphalt Mixture Image with Slit Island Method. J. Transp. Inf. Saf..

[42] Fukunaga A., Burns J.H.R., Craig B.K., Kosaki R.K. (2019). Integrating Three-Dimensional Benthic Habitat Characterization Techniques into Ecological Monitoring of Coral Reefs. J. Mar. Sci. Eng..

[43] Mancuso F., Milazzo M., Sarà G., Chemello R. (2023). Bi- and three-dimensional fractal analysis of the brown seaweed Gongolaria montagnei and their relationship with gastropod molluscs assemblage. Mar. Pollut. Bull..

[44] Zhou R., Luo Y., Fenster A., Spence J.D., Ding M. (2018). Fractal dimension based carotid plaque characterization from three-dimensional ultrasound images. Med. Biol. Eng. Comput..

[45] Free S.L., Sisodiya S.M., Cook M.J., Fish D.R., Shorvon S.D. (1996). Three-Dimensional Fractal Analysis of the White Matter Surface from Magnetic Resonance Images of the Human Brain. Cereb. Cortex.

