# Supplementary material for: 3D Morphological Scanning and Environmental Correlates of Bufo gargarizans in the Yellow River Basin
Source: Animals (Basel). 2024 Jan 24;14(3):369. doi: 10.3390/ani14030369 (PMC10854707; doi:10.3390/ani14030369)
Supplement: Supplementary file 1 [file animals-14-00369-s001.zip › animals-2795177-supplementary.pdf]

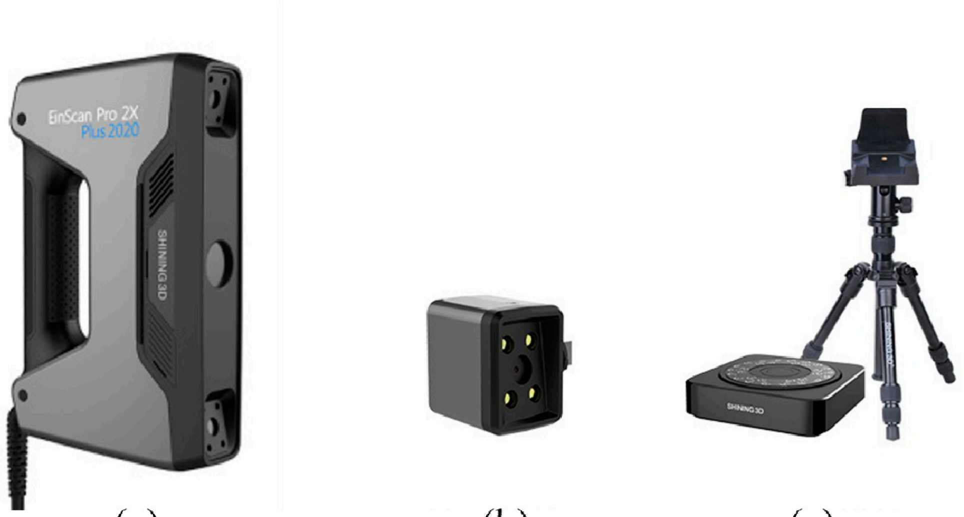

(a) (b) (c)

Figure S1. Three-dimensional scanner EinScan Pro 2X Plus 2020 work modules. (a) Handheld Module (b) Texture Module (c) Engineering Module, Stepper Motorized Rotary Table Triangle Stand.

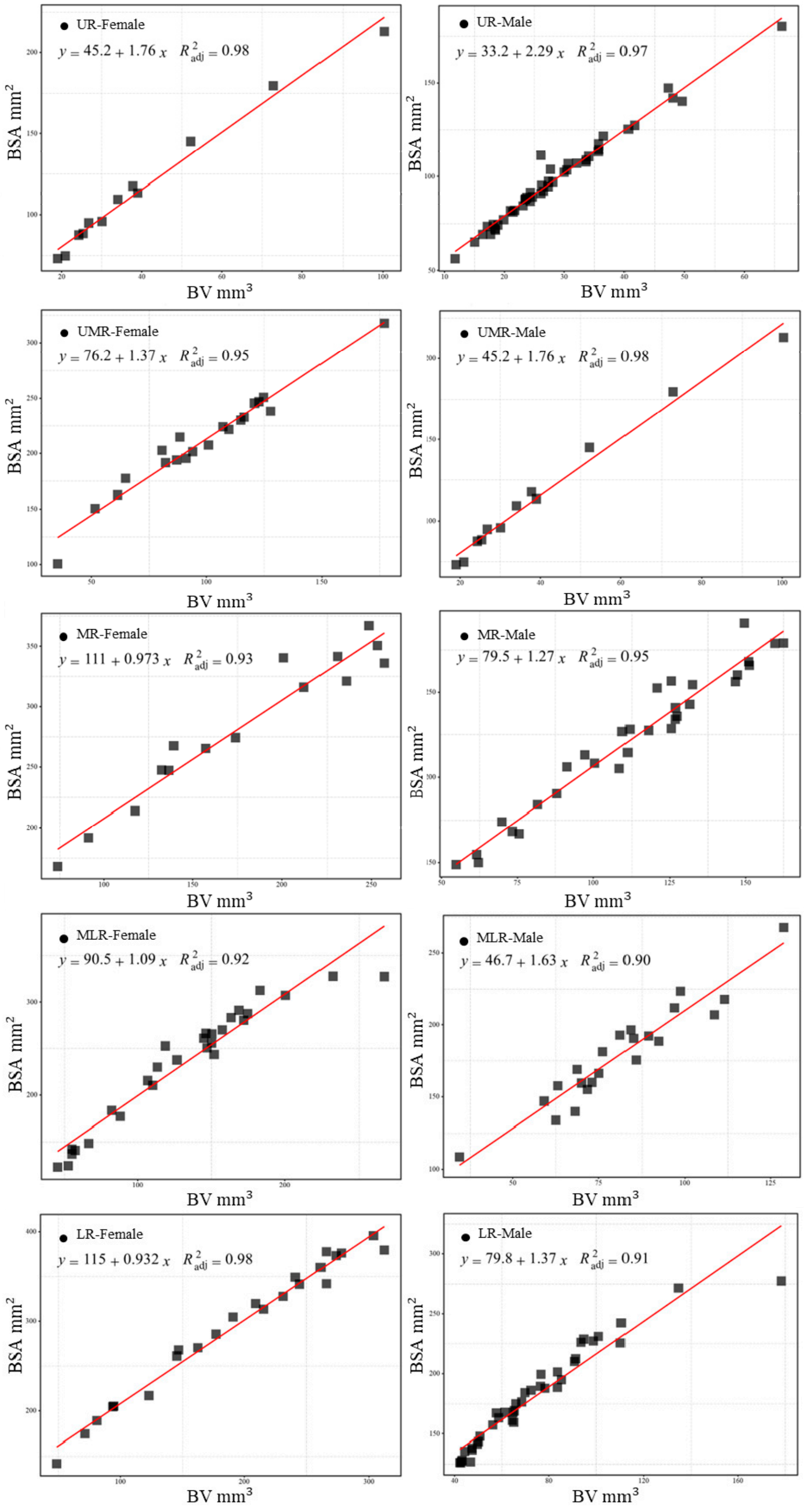

Figure S2. Relationship between body surface area and volume in females (left) and males (right) of different geographic populations of *B. gargarizans* in the Yellow River Basin.

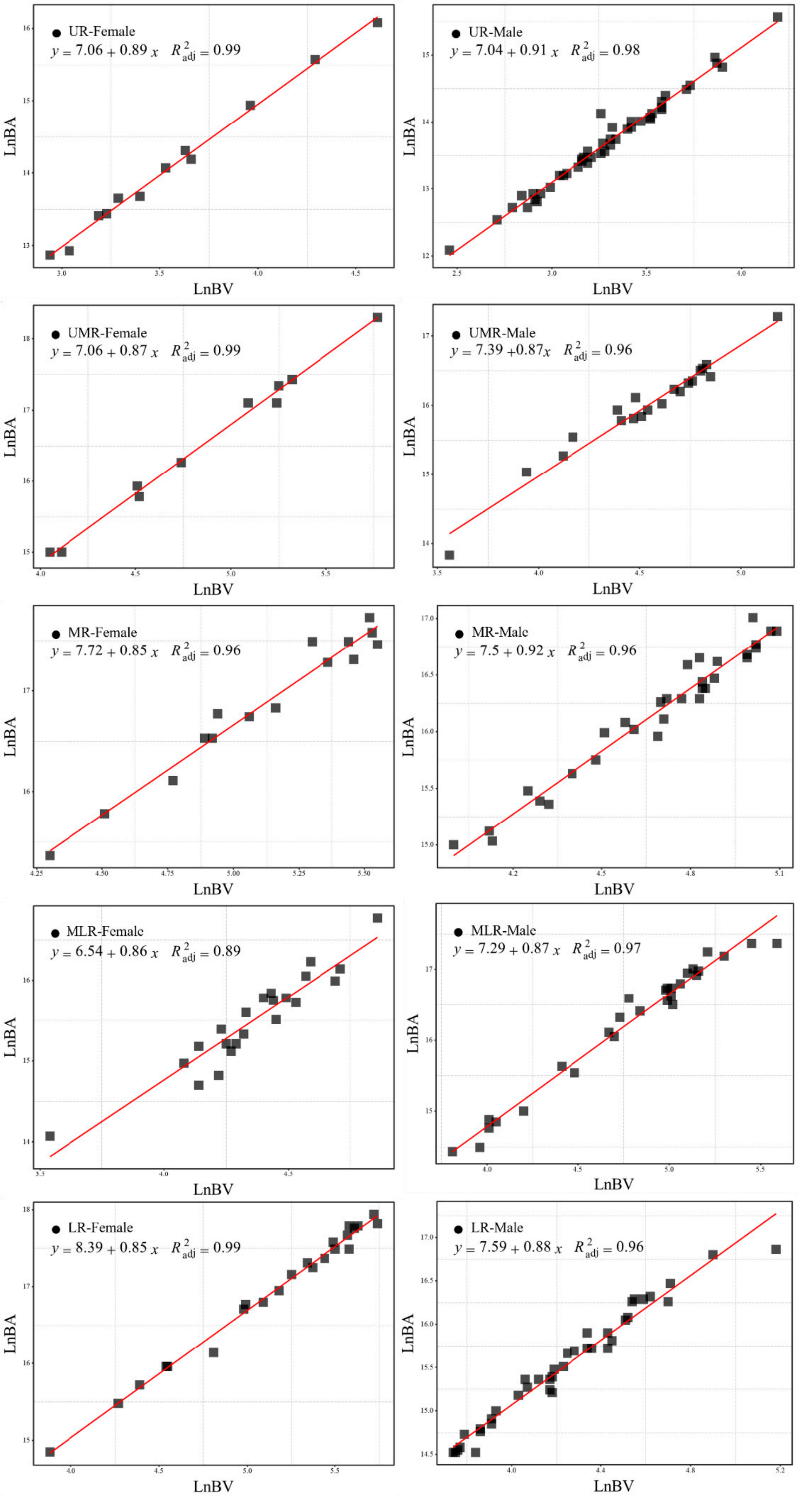

Figure S3. 3D fractal dimension map of females (left) and males (right) in different geographical populations of *B. gargarizans* in the Yellow River Basin.

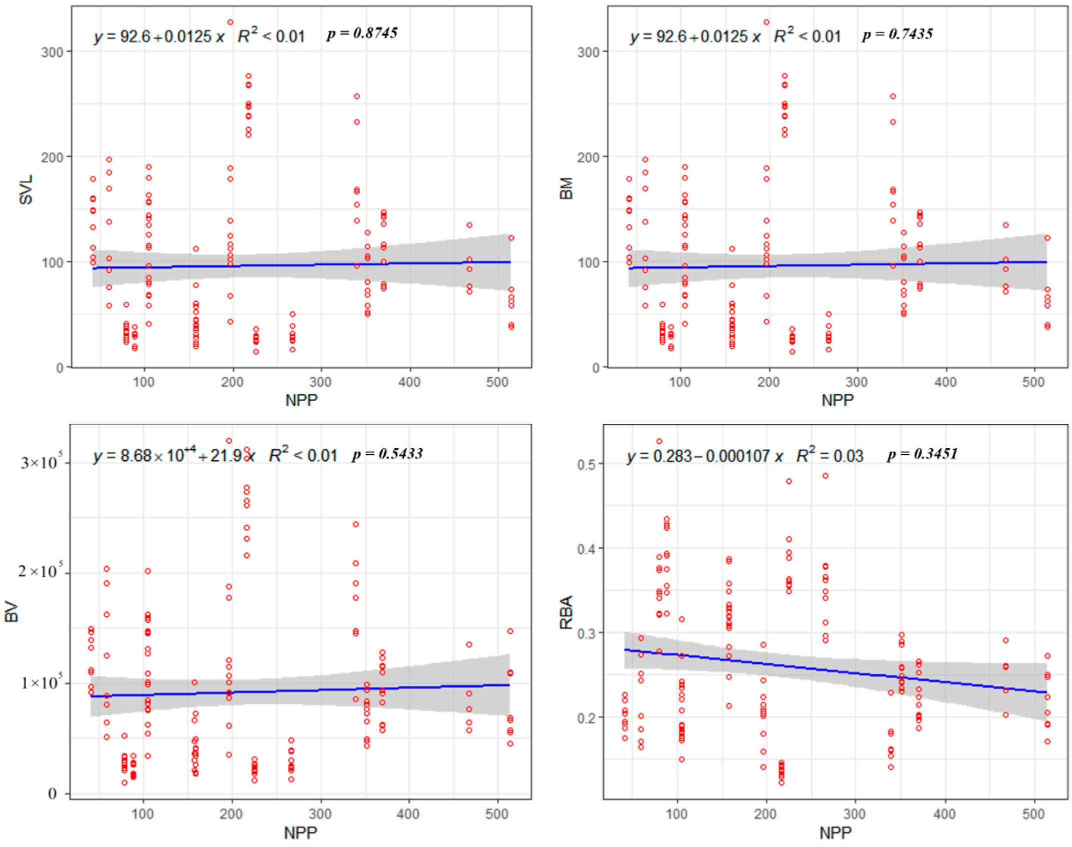

Figure S4. Linear regression modeling of NPP with SVL, BM, BV and RBA.
